# The Small GTPase RhoB Regulates TNFα Signaling in Endothelial Cells

**DOI:** 10.1371/journal.pone.0075031

**Published:** 2013-09-26

**Authors:** Jeffrey Kroon, Simon Tol, Sven van Amstel, Judith A. Elias, Mar Fernandez-Borja

**Affiliations:** Department of Molecular Cell Biology, Sanquin Research and Landsteiner Laboratory, University of Amsterdam, Amsterdam, The Netherlands; Baylor Institute for Immunology Research, United States of America

## Abstract

The inflammatory response of endothelial cells triggered by cytokines such as TNFα and IL1β plays a pivotal role in innate immunity. Upon pro-inflammatory cytokine stimulation, endothelial cells produce chemokines and cytokines that attract and activate leukocytes, and express high levels of leukocyte adhesion molecules. This process is mediated by intracellular signaling cascades triggered by activation of e.g. the TNFα receptor (TNFR) that lead to the activation of the NFκB transcription factor and of MAP kinases, which in turn activate inflammatory gene transcription. We found that the small GTPase RhoB was strongly and rapidly upregulated in primary human endothelial cells by TNFα, IL1β and LPS. We subsequently investigated the role of RhoB in the regulation of TNFR signaling in endothelial cells by silencing RhoB expression with siRNA. We provide evidence that the TNFα-induced activation of p38 MAP kinase is strongly dependent on RhoB, but not on RhoA, while JNK activation is regulated by both RhoB and RhoA. Consistent with the important role of p38 MAP kinase in inflammation, we demonstrate that loss of RhoB impairs TNFα-induced ICAM-1 expression and reduces cell production of IL6 and IL8. In addition, we show that RhoB silencing alters the intracellular traffic of TNFα after endocytosis. Since RhoB is a known regulator of the intracellular traffic of membrane receptors, our data suggest that RhoB controls TNFα signaling through the regulation of the TNFR traffic.

## Introduction

Tumor necrosis factor α (TNFα) is a pleiotropic pro-inflammatory cytokine that plays a pivotal role in the innate immune response to infection and tissue injury. Vascular endothelial cells respond to TNFα by upregulating the expression of cytokines and chemokines, such as IL-6 and IL-8, and of endothelial leukocyte adhesion molecules, such as VCAM-1, ICAM-1 and E-selectin [1]. These molecules enable TNFα-activated endothelial cells to attract, activate and recruit circulating leukocytes, which subsequently extravasate to reach the site of infection or injury. The inflammatory program induced by TNFα is a result of intracellular signaling triggered by the TNFα-receptor (TNFR) [2], [3]. Upon ligand binding, TNFR trimerizes and recruits TRAF-2 (TNFR-associated factor 2) and RIP1 (receptor interacting protein 1) to its cytoplasmic death domain. The formation of this signaling complex leads to the activation of the transcription factor NFκB and of the MAP kinases JNK and p38. Subsequently, the TNFR is rapidly endocytosed and eventually degraded in the lysosomes [4], [5]. However, TNFR internalization is clearly not only a mechanism of receptor downregulation but also of signaling compartmentalization, providing temporal and spatial regulation of the diverse signaling cascades triggered by the activated receptor [6]. While signaling from the TNFR leading to NFκB activation takes place at the plasma membrane, there is compelling evidence that TNFR pro-apoptotic signaling occurs on endosomes [4], [7]. In addition, several molecules involved in TNFR signaling are found on the surface of endosomal and lysosomal compartments [8], [9]. Finally, one study has demonstrated that internalization of the TNFR from the plasma membrane is a required step for the activation of p38 and JNK MAP kinases [10].

RhoB is a short-lived Rho GTPase whose expression is inducible by a variety of stimuli including growth factors, such as EGF and PDGF [11] and stress stimuli such as DNA-damaging drugs, UV irradiation and reactive oxygen species [12], [13]. RhoB is 83% identical to RhoA, a constitutively expressed GTPase and a well-established regulator of actomyosin-based contractility and of serum-induced transcription. Although these two GTPases bind to a similar set of proteins in solution, their non-overlapping intracellular distribution provides specificity to their respective actions [14]. Whereas RhoA is cytosolic and translocates to the plasma membrane upon activation, RhoB localizes to endosomes/multivesicular bodies [15]. Multivesicular bodies are primarily involved in the sorting of membrane proteins for their delivery to lysosomes for degradation. Consistently, RhoB regulates the sorting and degradation of growth factor and cytokine receptors [16]–[21]. In agreement with the role of Rho GTPases as critical regulators of actin dynamics, RhoB appears to control vesicle traffic through the regulation of actin polymerization on endosomes [22], [23], possibly through the recruitment and activation of Diaphanous proteins [23], [24].

Inflammatory cytokines such as tumor necrosis factor α (TNFα and interleukin 1β (IL1β activate endothelial cells by inducing multiple intracellular signaling pathways that regulate gene expression. The small GTPase RhoB is a short-lived protein encoded by an immediate-early gene that is rapidly activated in response to a wide variety of stimuli including growth factors, UV radiation and oxidative stress [11]–[13]. Here we report that RhoB protein is rapidly upregulated in primary human endothelial cells by TNFα, IL1β and bacterial lipopolysaccharide (LPS). We have addressed the role of RhoB in TNFR signaling by using siRNA-mediated knockdown of RhoB. We present evidence that RhoB is essential for the activation of p38 and JNK MAP kinases, but not NFκB, by TNFα. Finally, we show that RhoB silenced cells accumulate endocytosed TNFα pointing to a defect in traffic kinetics and/or receptor sorting. In summary, our data suggest that RhoB has as role in TNFα signaling through the regulation of TNFR intracellular traffic.

## Materials and Methods

### Cell culture

Pooled primary human umbilical vein endothelial cells (HUVEC, Lonza) were seeded on fibronectin-coated culture flasks and maintained in EGM-2 medium (Lonza) in a humidified atmosphere of 95% air and 5% CO2 at 37°C.

### Reagents and antibodies

Recombinant human TNFα, IL1β, IFNγ, VEGF and TGFβ were from R&D Systems. Bacterial lipopolysaccharide (LPS) was from Sigma.The pharmacological inhibitors cycloheximide, MG132 and N-acetyl-cysteine (NAC) were from Sigma. SC-514, SP600125 and SB230580 were from Calbiochem. Rabbit and mouse anti-RhoB, mouse anti-RhoA, mouse anti-phospho-ERK, mouse anti-phospho-JNK, goat anti-VCAM-1 and mouse anti-NFκB p50 were obtained from Santa Cruz Biotechnology. Rabbit anti-phospho-p38 and mouse anti-IκBα were obtained from Cell Signaling. Mouse anti-α-tubulin (DM1A) was from Sigma. Mouse anti-EEA1 and mouse anti-RhoGDI were from BD Biosciences. Secondary antibodies labeled with Alexa488 or Alexa568 for immunofluorescence were purchased from Invitrogen. Secondary antibodies labeled with horseradish peroxidase for immunoblotting were from Amersham.

### Immunoblotting and phosho-MAP kinase arrays

Cells were lysed in cold NP-40 buffer (1% NP40, 100 mM NaCl, 100 mM MgCl_2_, 10% glycerol, 50 mM Tris pH 7.4) containing a cocktail of phosphatase and protease inhibitors (Thermo Scientific). Protein content of lysates was quantified with the Precision Red Advanced Protein Assay Reagent (Cytoskeleton). Equal protein concentrations were loaded in SDS-PAGE gels and analyzed by Western blotting. Equal loading was additionally controlled by detection of Rho-GDI and αtubulin. The pixel density of each band was determined with ImageJ and values corrected by the corresponding αtubulin intensities. These values were normalized to those of siRNA control-transfected cells. Non-parametric one-way ANOVA Tukey test was used to evaluate statistical significance of at least 3 independent experiments. A two-way ANOVA with Bonferroni post-test was used to evaluate statistical significance when the effects of several siRNA effects were compared at different time points on TNF stimulation. Results are expressed as mean ± SEM (*p<0.05; **p<0.01; ***p<0.001).

Human phospho-mitogen activated kinase protein antibody arrays were purchased from R&D Systems and used according to manufacturer instructions. Briefly, cell lysates were incubated with the antibody arrays overnight at 4°C. After washing, arrays were incubated with a mixture of phospho-site specific biotinylated antibodies for 2 hours at room temperature. Bound biotinylated antibodies were detected with HRP-streptavidin. Arrays were developed in ECL and exposed to X-Omat films. Digital scans of the films were analyzed for pixel density with ImageJ. Averaged background values corresponding to the negative controls were subtracted from the values of each spot. Values for duplicates on the array were averaged and represented as a percentage of the pixel density of the positive controls included in the array.

### RhoB activity assay

Rho-GTP pulldown assays were performed as previously described [25]. Cells were lysed on ice in lysis buffer containing 50 mM Tris pH 7.6, 500 mM NaCl, 1% Triton X-100, 0.1% SDS, 0.5% deoxycholate, 10 mM MgCl_2_, 100 and a cocktail of protease inhibitors (Sigma). Lysates were clarified by centrifugation at 14,000×g 5 min and incubated with Glutathion S-transferase (GST)-Rho binding domain beads. After washing four times in 50 mM Tris pH 7.6, 150 mM NaCl, 1% Triton X-100, 0.5 mM MgCl_2_, 100 µM orthovanadate, with protease inhibitors, bound Rho proteins were solubilized with SDS-sample buffer and analyzed by SDS-PAGE with specific antibodies for RhoB.

### RhoB and RhoA knock-down with siRNA

Cells were transfected with a pool of 3 siRNA duplexes for RhoB (sc-29472, Santa Cruz Biotechnology, 5′-3′ CCCUUGUCUGUAACAUAGAAs(siRhoB#1); CCACACUUGUACGCUGUAA(siRhoB#2); CCAGUGGUACUUCUACUAA(siRhoB#3) or with either siRNA#1 or #2. siRNA duplex for RhoA (sc-29471, Santa Cruz Biotechnology, 5′-3′ GGCAGAGAUAUGGCAAACA). As control we used a non targeting 20–25 nucleotide siRNA designed as a negative control (Control siRNA-A, sc-37007, Santa Cruz Biotechnology). Transfection of HUVEC was performed using the siRNA transfection reagent and medium from Santa Cruz Biotechnology according to the manufacturer's protocol. Briefly, HUVEC were seeded the day previous to transfection in EGM-2 without antibiotics. For transfection, cells were washed in transfection medium and incubated with a mixture of siRNA and transfection reagent for 5 hours at 37°C and 5% CO_2_. Cells were then rinsed and incubated for a period of 48 to 72 hours before stimulation and analysis.

### TNFα endocytosis

To analyse TNFα endocytosis and intracellular traffic, cells were incubated with biotin-labelled human TNFα (R&D Systems) for 1 hour at 4°C followed by 30 minutes incubation with FITC-avidin (R&D Systems) also at 4°C. After washing, cells were either immediately fixed in 3.7% formaldehyde or transferred to 37°C to allow TNFα internalization and fixed after different incubation times for immunofluorescence analysis. Flow cytometry analysis was performed as indicated above but cells were detached from the dish with trypsin-EDTA (Lonza) after each incubation time. After addition of Trypsin Neutralizer Solution (Lonza), cells were kept on ice. Flow cytometry analysis of FITC-positive cells was performed in a FACS Canto (BD Biosciences).

### Immunofluorescence

Cells seeded on fibronectin-coated glass coverslips were fixed in formaldehyde 3.7%, permeabilized with 0.1% Triton X-100 and blocked with PBS containing 0.5% bovine serum albumin (PBS-BSA). Primary antibodies were incubated for 1 hour at room temperature followed by 30 minutes incubation with secondary antibodies. All antibodies were diluted in PBS-BSA. Images of stained cells were collected with a LSM510 confocal microscope (Zeiss). Images were analyzed for quantitative co-localization using Zen 2009 software (Zeiss).

### Cytokine ELISA

Cytokine levels of IL6 and IL8 were measured in the supernatants of HUVEC transfected with Control, RhoB or RhoA siRNAs and stimulated with TNFα for 4 and 16 hours using commercially available enzyme-linked immunosorbent assay (ELISA) kits (PeliKine Compact™ human ELISA kits, Sanquin, Amsterdam, The Netherlands) as previously described [26]. The plates were read in an ELISA reader (Labsystems Multiskan Multisoft, Helsinki, Finland) at 450 nm, with 540 nm as a reference.

### Statistical analyses

Statistically significant differences between data means were determined by two-tailed paired Student's *t* test using Excel software (Microsoft); *P*<0.05 was considered statistically significant.

## Results

**Figure 1:**
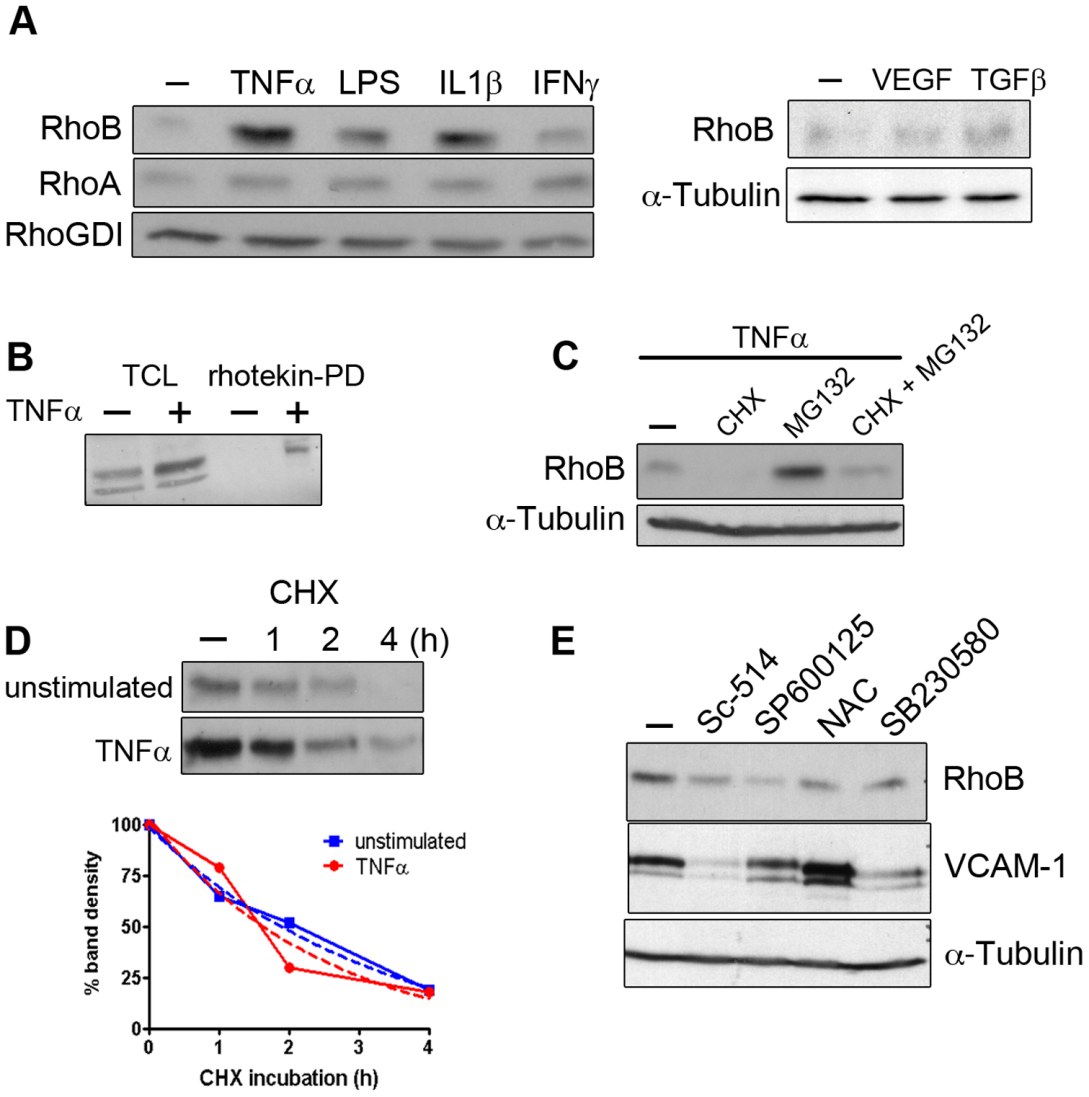
Pro-inflammatory mediators induce ‘de novo’ RhoB synthesis and RhoB activation in human umbilical vein endothelial cells. (A) Lysates of HUVEC incubated for 16 h with the indicated stimuli were analyzed for the expression of RhoB and RhoA by western blot. RhoGDI and tubulin were detected to control for equal loading; (B) Pull-down of GTP-Rho from HUVEC stimulated or not with TNFα for 4 h. Precipitates were analyzed for the presence of RhoB; (C) RhoB expression was induced by a 4 h TNFα stimulation, and subsequently proteasome inhibitor MG132 and/or the protein synthesis inhibitor cycloheximide was added to the cells for an additional 4 hours incubation; (D) RhoB detection in lysates of HUVEC stimulated with TNFα for 4 hours before the addition of cycloheximide (CHX) for 1, 2 or 4 hours. A digital scan of the film was made and the intensity of the RhoB bands was measured using ImageJ software. Data are shown as percentage of the RhoB present in the absence of cycloheximide. Unstimulated (solid blue line) and TNFα-stimulated cells (solid red line). Fitted regression lines obtained by linear regression analysis are shown as dotted lines; (E) Endothelial cells were incubated with TNFα in combination with the NFκB inhibitor sc-514, the JNK inhibitor SP600125, the p38 inhibitor SB230580 or the anti-oxidant N-acetyl cysteine (NAC). RhoB and VCAM-1 were detected in cell lysates. α-Tubulin was detected to control for equal loading.

We found that the pro-inflammatory mediators TNFα, IL1β and bacterial LPS (lipopolysaccharide) potently stimulated RhoB expression in primary human endothelial cells, while RhoA expression was unchanged (Figure 1A). In contrast, other endothelial stimuli such as interferon γ (INFγ, transforming growth factor β (TGFβ and vascular endothelial growth factor (VEGF) had little effect on RhoB expression (Figure 1A).

To assess whether, in addition to RhoB protein levels, TNFα also increases the levels of activated RhoB, we precipitated GTP-Rho with the Rho-binding domain of rhotekin coupled to Sepharose beads [25] and detected RhoB by western blotting. GTP-RhoB could be detected in lysates of cells stimulated with TNFα (Figure 1B), which indicates that TNFα increases the level of activated RhoB in endothelial cells.

Next, we sought to determine the mechanism by which TNFα induced RhoB up-regulation. Two mechanisms regulate RhoB levels in cells: increased synthesis and protein stabilization [12], [27]–[29]. To discriminate between these two possibilities, we used inhibitors of protein synthesis (cycloheximide) and of proteasomal degradation (MG132). We incubated HUVEC with TNFα alone for 4 hours, in order to accumulate RhoB, and subsequently added cycloheximide and/or MG132 for an additional 2 hours. Cycloheximide incubation caused the complete depletion of RhoB whereas MG132 increased RhoB levels when compared to cells stimulated with TNFα alone (Figure 1C). These results indicate that TNFα upregulates RhoB protein synthesis and that newly synthesized RhoB is rapidly degraded by the proteasome. Consistently, inhibition of both synthesis and degradation after TNFα stimulation resulted in RhoB levels comparable to those in unstimulated cells (Figure 1C). To estimate the half-life of RhoB in resting and in TNFα-stimulated cells, the kinetics of RhoB degradation was examined. Endothelial cells were first stimulated with TNFα for 4 hours to accumulate RhoB in cells. Then, protein synthesis was inhibited by the addition of cycloheximide for 1, 2 and 4 hours and RhoB levels were analyzed by western blotting. A progressive loss of RhoB was observed correlating with the duration of cycloheximide treatment in both control and TNFα-treated cells (Figure 1D). We plotted the band intensity as a percentage of the RhoB present in the absence of cycloheximide and fitted the data points using a one-phase exponential decay function (Figure 1D). The estimated values for the half-life of RhoB was 2.3 hours in unstimulated cells and 1.7 hours in TNFα-stimulated cells. Thus, TNFα does not significantly change the half-life of RhoB, suggesting that TNFα does not enhance RhoB protein stability.

TNFα activates gene transcription through the activation of both NFκB and MAP kinase pathways [3]. To assess the involvement of these pathways in the upregulation of RhoB by TNFα, we tested different pharmacological inhibitors of NFκB and p38 and JNK MAP kinases [30]. Although the inhibitor of NFκB, sc-514 and the ROS scavenger N-acetyl-cysteine (NAC) impaired RhoB induction by TNFα, the largest effect was observed with the JNK inhibitor SP600125 (Figure 1E).

**Figure 2:**
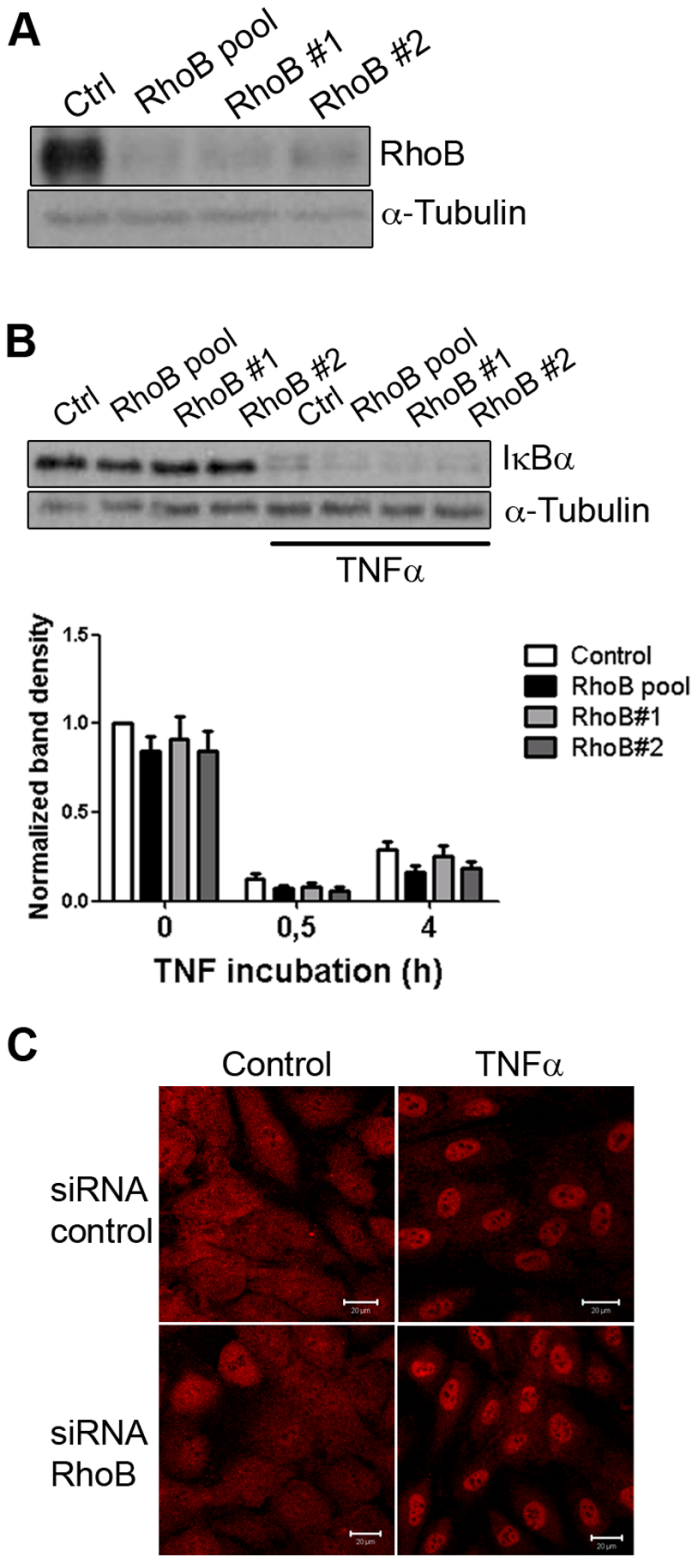
RhoB does not regulate TNFα-induced NFκB activation. (A) Efficiency of RhoB silencing with either a pool of 3 siRNAs (RhoB pool) or with two single siRNAs from the pool individually (#1 and #2) was analyzed in cells stimulated with TNFα for 16 hours; (B) TNFα was added for 30 min to cells transfected with RhoB siRNAs as in (A) or with an siRNA control. Cell lysates were analyzed for the presence of IκBα. α-Tubulin was detected to control for equal loading. Graphs represent band densities corrected for protein loading and are normalized to control transfected cells. No significant differences were found between control and RhoB-deficient cells; (C) Endothelial cells transfected with siRNA control or with a pool of RhoB siRNAs were stimulated with TNFα for 0 and 30 min, fixed and permeabilized and then incubated with an antibody to the p50 NFκB subunit.

To explore the role of RhoB in TNFα-induced inflammation we examined the two main signaling pathways triggered by TNFα,; NFκB and MAPK, after silencing RhoB expression using siRNA-mediated knock-down with a pool of 3 different siRNAs or with single siRNAs (#1 and #2) from this pool individually (Figure 2A). Activation of NFκB by TNFα results in the phosphorylation and subsequent degradation of inhibitory IκB proteins [3]. Neither RhoB, nor RhoA downregulation affected the breakdown of the IκBα chain following TNFα stimulation (Figure 2B and Figure S1A). Consistent with these results, RhoB silencing did not prevent NFκB nuclear translocation, as determined by immunofluorescent staining for the p65 NFκB subunit (Figure 2C). Thus, RhoB does not regulate NFκB activation by TNFα.

**Figure 3:**
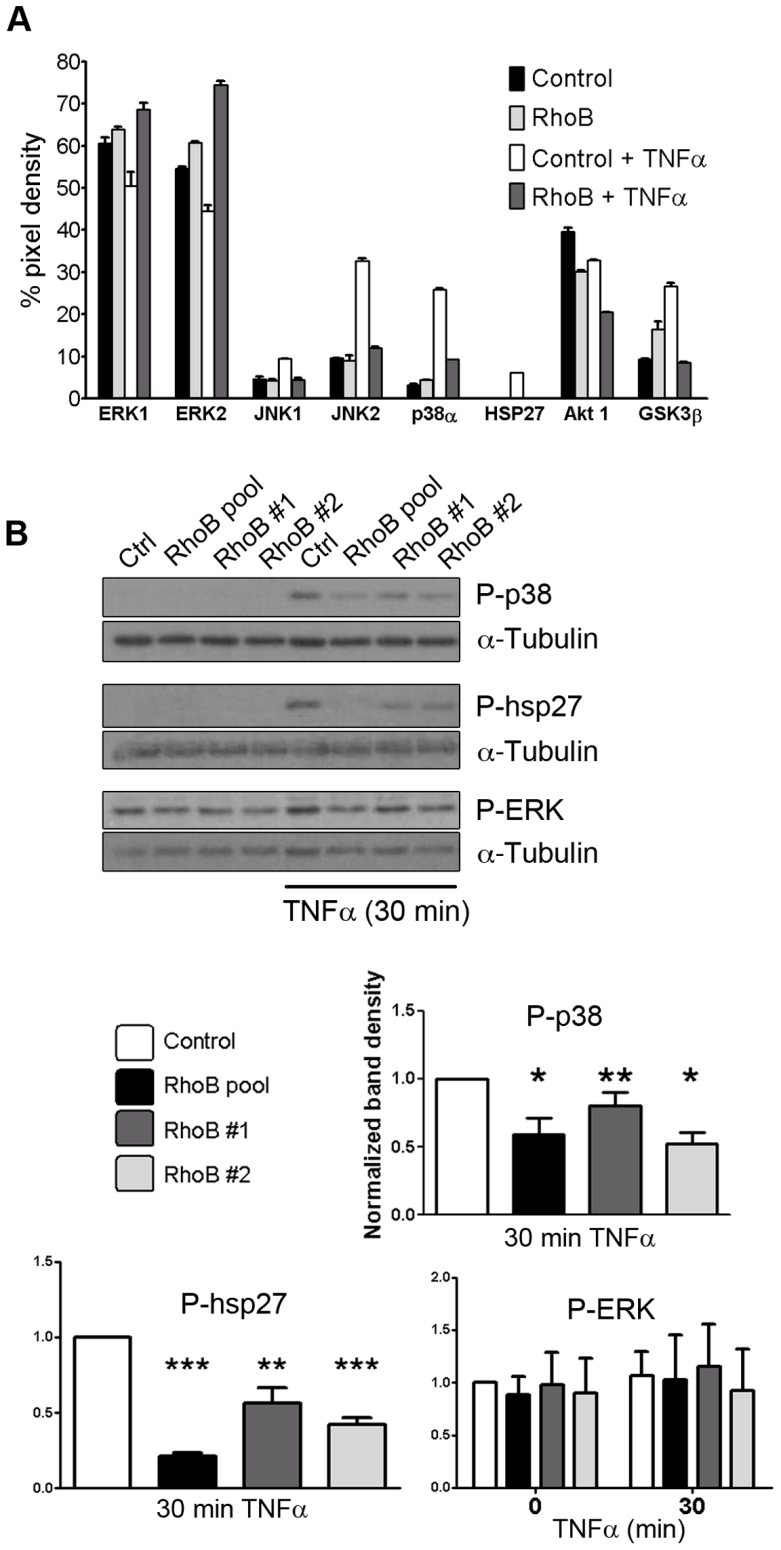
RhoB regulates MAP kinase activation by TNFα. (A) Cells transfected with siRNA control or with a pool of RhoB siRNAs were stimulated or not with TNFα for 30 min. Lysates were prepared and incubated with an anti-phospho-MAP kinase antibody array. Pixel intensity of spots in the array was determined, corrected for background and represented as percentage of the positive controls included in the array; (B) Western blot analysis of phospho-ERK1/2, phospho-p38 and phospho-hsp27 in HUVEC transfected with siRNA control, with a pool of RhoB siRNAs (RhoB pool) or with single RhoB siRNAs (RhoB#1 and #2).

We next used anti-phospho MAP kinase antibody arrays to analyze changes in phosphorylation of various cellular serine/threonine kinases in endothelial cells transfected with RhoB siRNA or control siRNA before and after TNFα stimulation. The results of this analysis showed that TNFα-induced phosphorylation of JNK (JNK1, JNK2), p38α and heat shock protein 27 (hsp27, a substrate of the p38 MAP kinase pathway) is abrogated in RhoB-deficient cells (Figure 3A). In support of the validity of the MAP kinase array analysis, RhoB silencing also reduced the levels of phosphorylated Akt and GSK3β, as previously described in endothelial cells [31] and in keratinocytes [32]. We further confirmed the effects of RhoB silencing on the levels of phosphorylated MAP kinases by traditional western blotting. Similar to the results with phospho-MAP kinase arrays, RhoB knock-down resulted in decreased levels of phospho-JNK, phospho-p38 and phospho-hsp27 upon TNFα stimulation (Figure 3B and Figure S1B). In contrast, RhoB silencing had no effect on ERK activation, indicating that RhoB only regulates stress-activated MAPKs. To test for the specificity of RhoB in p38 activation, we examined p38 activation in RhoA-silenced cells (Figure S1B). These data suggest that both RhoB and RhoA are required for TNFα-induced JNK activation while RhoB specifically regulates p38 activation. Interestingly, we found that blocking RhoA expression upregulates the cellular levels of RhoB by approximately 6-fold in unstimulated and 3-fold in TNFα-stimulated cells. RhoB knock-down only moderately increases RhoA levels by less than 2-fold.

**Figure 4:**
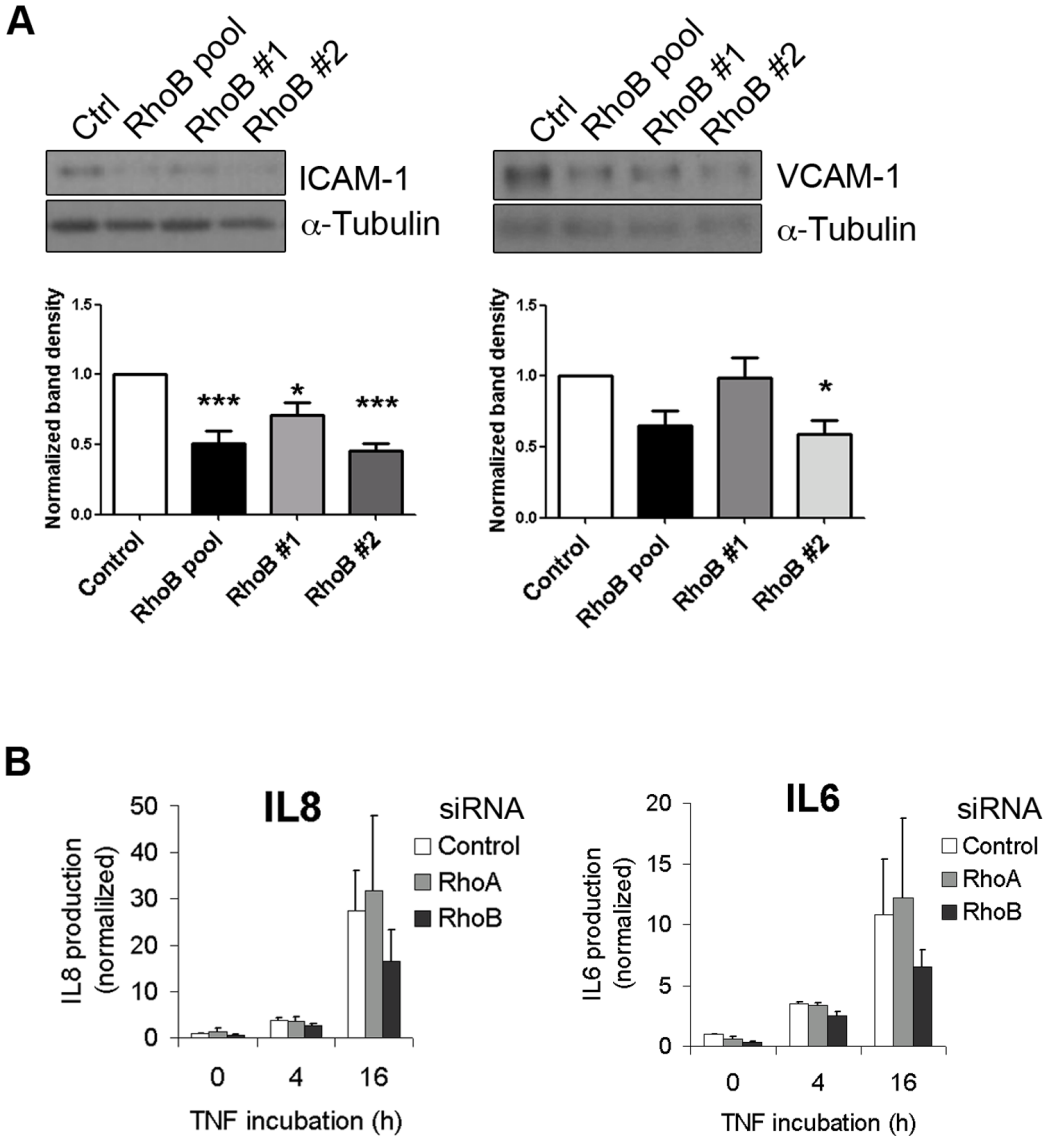
RhoB silencing impairs TNFα-induced pro-inflammatory molecule expression. (A) Lysates of cells transfected with siRNAs as in Figure 3 were stimulated with TNFα for 4 h and analyzed for total ICAM-1 and VCAM-1 expression by western blotting; (B) ELISA analysis of IL6 and IL8 present in conditioned media of cells transfected with a pool of RhoB siRNAs, with RhoA siRNA or a control siRNA were stimulated with TNFα for 4 or 16 h. Graph shows normalized values after dividing by the IL concentration in the medium of unstimulated siRNA control-transfected cells (1235±592 pg/mL IL8 and 187±7.5 pg/mL IL6) (mean ± SEM, n = 3; *p<0,05).

MAPKs p38 and JNK regulate TNFα-induced pro-inflammatory gene expression [33], [34]. Since our data show that RhoB regulates MAPK activation by TNFα, we next tested whether RhoB is required for the pro-inflammatory response of endothelial cells. To this end, we analyzed the expression of VCAM-1 and ICAM-1, as well as of the production of IL8 and IL6 (Figure 4). RhoB silencing significantly reduced TNFα-induced ICAM-1 expression. Although VCAM-1 levels appeared slightly reduced, the changes were not statistically significant (Figure 4A). In addition, RhoB silencing diminished the endothelial production of IL8 and IL6 (Figure 4B). Thus, RhoB appears to be required for optimal expression of pro-inflammatory molecules by endothelial cells upon TNFα stimulation.

**Figure 5:**
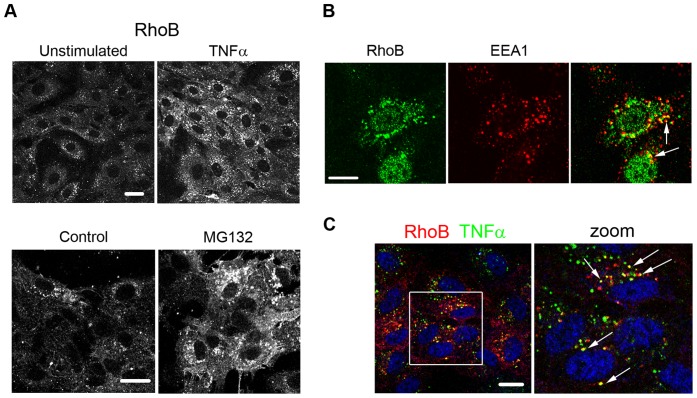
TNFα is internalized into RhoB-positive vesicles. (A) Cells were stained for RhoB before and after stimulation with TNFα for 4 hours (upper panels) and before and after proteasome inhibition with MG132 for 1 hour (lower panels); (B) Cells were stimulated with TNFα for 4 h and stained for RhoB (green) and EEA1 (red). Arrows point to vesicles positive for both proteins; (C) Cells were incubated at 4°C with biotin-TNFα and FITC-streptavidin (green) and transferred to 37°C for 30 min to allow internalization. Following fixation/permeabilization, cells were stained for RhoB (red). A magnification of the area within the box is shown on the right. Arrows point to vesicles where TNFα colocalises with RhoB. Bars: 10 µm.

RhoB modulates a variety of signal transduction pathways through the regulation of receptor traffic [16]–[21]., therefore we addressed the question whether RhoB also controls intracellular traffic of the TNFR. RhoB localized to vesicles in endothelial cells stimulated with TNFα (Figure 5A, upper panels). This distribution is similar to that found in unstimulated cells treated with proteasome inhibitor in order to accumulate RhoB to detectable levels (Figure 5A, lower panels). Thus, TNFα does not appear to change the subcellular distribution of RhoB, which localizes to EEA1-positive early endosomes (Figure 5B) [23], [35].

Upon TNFα binding, the TNFR is internalized into endosomes, which subsequently fuse with trans-Golgi network-derived vesicles, and is finally transported to lysosomes [4]. To address the question whether RhoB regulates TNFR traffic we used biotin-TNFα/FITC-streptavidin as previously described [4]. Cells were incubated at 4°C with biotin-TNFα followed by incubation with FITC-streptavidin. Subsequently, internalization of biotin-TNFα/FITC-streptavidin complexes was allowed by transfer of the cells to 37°C for various periods of time. Detection of RhoB shows that internalized TNFα colocalizes with RhoB (Figure 5C), suggesting that the TNFR traffics through RhoB-positive endosomes.

**Figure 6:**
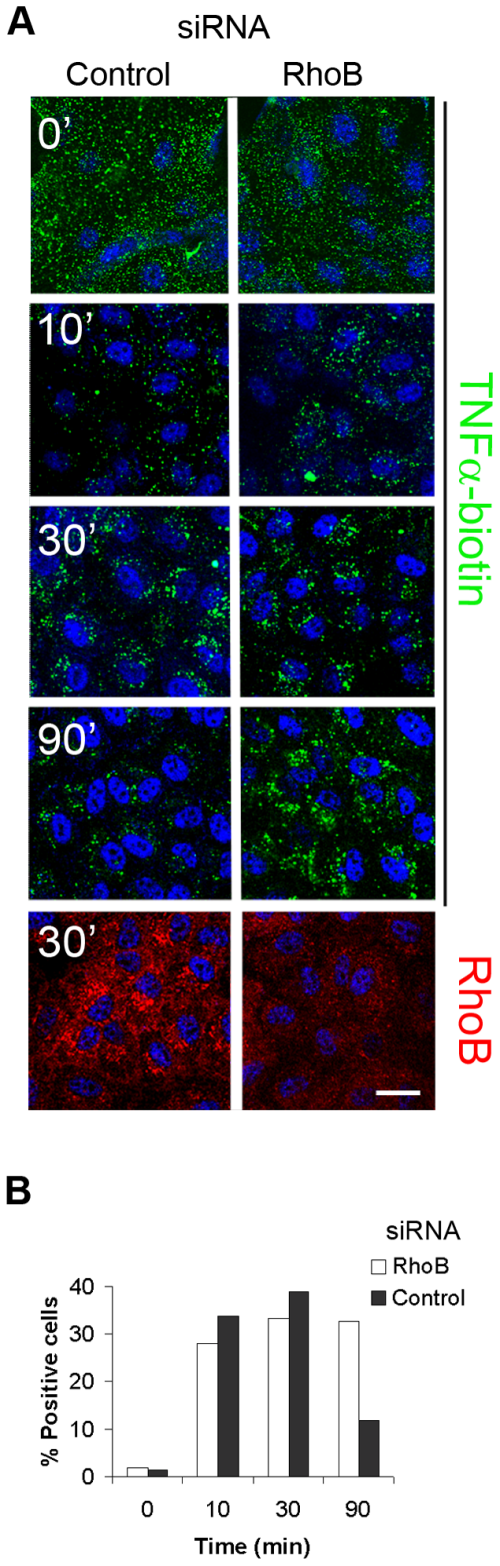
RhoB-silencing causes accumulation of internalized TNFα in cells. (A) Cells transfected with siRNA control or a pool of 3 RhoB siRNAs were incubated with biotin-TNFα and FITC streptavidin at 4°C and then transferred to 37°C for 10, 30 or 90 min. Cells were fixed/permeabilized and stained for RhoB. Images were obtained with a confocal microscope (lower panels). (B) Cells transfected and incubated with biotin-TNFα and FITC streptavidin as in (A) were detached with trypsin and FITC fluorescence were analyzed by flow cytometry. Bar: 20 µm.

We then studied TNFα internalization upon RhoB silencing by immunofluorescence (Figure 6A) and flow cytometry (Figure 6B) in a time-course experiment. No apparent differences were observed in the amount of biotin-labelled TNFα bound to the cell surface between control and RhoB-deficient cells, which indicates that RhoB does not control surface expression of the TNFR. Also after 10 and 30 minutes of internalization, similar intracellular amounts of TNFα were found in control and RhoB-negative cells (Figure 6A). However, after 90 minutes, RhoB-deficient cells contained a larger amount of TNFα than control cells (Figure 6A), suggesting that RhoB plays a role in TNFR traffic. We performed the same experiment and quantitatively analyzed cell-associated fluorescence by flow cytometry (Figure 6B). These results recapitulate those found by confocal microscopic analysis. The absence of TNFα-positive cells at time point 0 can be explained by the fact that cells are suspended by trypsinization, which is likely to cause the loss of membrane-bound TNFα.

## Discussion

Rho GTPases are key signaling components controlling the inflammatory response elicited by pro-inflammatory cytokines [36]. In our study, we examined the role of RhoB in the inflammatory response elicited by TNFα in primary human endothelial cells.

We show here that pro-inflammatory mediators such as TNFα, IL1β and LPS upregulate RhoB expression, whereas RhoA appears to be constitutively expressed. Our data suggest that TNFα increases RhoB protein synthesis without affecting protein stability. The half-life of RhoB in unstimulated endothelial cells was of ∼2 hours, as previously shown in other cell types, and of 1.7 hours in TNFα-stimulated cells, indicating that TNFα does not promote RhoB protein stabilization. Our data suggest that TNFα induces the transcriptional activation of the immediate-early gene encoding RhoB mainly through a JNK-dependent pathway, previously involved in the transcriptional upregulation of RhoB [37], [38].

Following our initial observation that TNFα potently stimulates RhoB protein synthesis and activity, we argued that RhoB might have a role in TNFα-dependent signaling. First, we show that TNFα increases active GTP-RhoB in endothelial cells. This increase may be a consequence of the increase in RhoB protein rather than of enhanced RhoB activation by TNFα-regulated guanine nucleotide-exchange factors (GEFs). Although the exchange factor that activates RhoB in endothelial cells is currently unknown, recent work showed that GEF-H1 mediates LPS-induced RhoB activation in dendritic cells [39]. Future studies will determine if GEF-H1 is also involved in RhoB activation by TNFα in endothelial cells.

The enhanced activity of RhoB in TNFα-stimulated cells suggested a role for RhoB in TNFR signaling. We studied the activation of the two main signaling cascades triggered by TNFα; the NFκB and MAPK pathways, after RhoB silencing with siRNA. To control for specificity of RhoB action, we silenced RhoA, a close member of the Rho GTPase subfamily of Ras GTPases. Our studies revealed that RhoB does not regulate NFκB activation by TNFα. However, activation of p38 MAP kinase by TNFα is critically dependent on RhoB, while both RhoB and RhoA are required for JNK activation. To the best of our knowledge, this is the first study on the specific role of endogenous RhoA or RhoB in the activation of p38 and JNK by TNFα in primary human endothelial cells. Our findings are supported by previous studies using Rho-targeting toxins or Rho mutants [40], [41]. Inhibition of all Rho isoforms (RhoA, B and C) with C3 toxin was shown to impair TNFα-induced p38 activation in endothelial cells [40]. In addition, expression of active mutants of RhoA and RhoB was shown to activate JNK in 293T cells [41]. Thus, RhoB regulates TNFα-dependent activation of stress-activated MAPKs in endothelial cells.

Given the central role that JNK and p38 play in inflammatory responses elicited by TNFα, our findings led to the hypothesis that RhoB has a pro-inflammatory role in endothelial cells. Further proof for this hypothesis was obtained upon examination of various pro-inflammatory proteins regulated by the p38 and/or JNK pathways. We show that RhoB silencing impairs TNFα-induced production of IL6 and IL8 and significantly reduces the expression of ICAM-1. Consistently, TNFα-induced activation of p38 was previously found to be critical for the expression of pro-inflammatory molecules such as TNFα, IL6, IL8 and cyclooxygenase-2 (COX-2) [42]–[48]. Similarly, p38 was previously shown to regulate ICAM-1 expression in endothelial cells [33], [34]. Collectively, our data strongly suggest that RhoB participates in the pro-inflammatory response of endothelial cells to TNFα through the regulation of p38 activation.

RhoB is an endosomal GTPase that regulates endosome dynamics through the recruitment of actin-polymerizing proteins of the formin family [23], [24]. Accordingly, RhoB controls the endocytic traffic and signaling of growth factor and chemokine receptors [16]–[21]. We assessed whether RhoB also regulates TNFR traffic by analyzing TNFα internalization in RhoB-deficient cells. Although we could not detect the TNFR by immunofluorescence due to high background and low specific signal of anti-TNFR antibodies, we are confident the intracellular traffic of TNFα reflects that of TNFα/TNFR complexes [4]. First, we show that RhoB localizes to endosomes and that internalized TNFα traffics through RhoB-positive endosomes. RhoB silencing causes the intracellular accumulation of endocytosed TNFα whereas, in control cells TNFα disappears in time. These data show that RhoB is involved in the regulation of the intracellular trafficking of TNFα and suggest that RhoB is required for the sorting of the TNFR to the degradative pathway, in a similar manner as previously described for the CXCR2 receptor [19].

Even though we do not yet have direct proof, we speculate that RhoB participates in the activation of MAPKs by TNFα through the regulation of TNFR traffic. In support of our hypothesis, EEA-1 positive endosomes carry activated MAP kinases [49], [50] and inhibition of receptor endocytosis hampers downstream activation of these kinases, suggesting that kinase activation takes place in an intracellular endocytic compartment [51]. Specifically, TNFR internalization is required for the activation of MAPK and Akt but not for IκBα degradation [10], suggesting that TNFR activates MAP kinases from an intracellular compartment whereas NFκB activation occurs at the plasma membrane. Similarly, TNFR-induced caspase activation takes place on endosomes [4]. Finally, endocytic compartments have been involved in the TNFα-dependent expression of cytokines and adhesion molecules [8], [52].

In summary, our study shows that RhoB is critically required for the inflammatory response of endothelial cells to TNFα, likely through MAP kinase activation downstream of the TNFR. In addition, our data suggest that RhoB may regulate TNFR signaling through its regulation of TNFR endocytic traffic kinetics and/or of receptor sorting.

## Supporting Information

Figure S1 (A) Cells transfected with a pool of 3 RhoB siRNAs, with a RhoA siRNA or with siRNA control were stimulated or not with TNFα for 30 minutes and IκBα was detected by western blotting of cell lysates. α-Tubulin was detected as control for equal protein loading; (B) Cells transfected with siRNAs mentioned above were stimulated with TNFα for 30 minutes. Subsequently, phospho-p38 and phospho-JNK were detected by western blotting of cell lysates. α-Tubulin was detected as control for equal protein loading.
(TIF)

## References

[1] Bradley JR (2008) TNF-mediated inflammatory disease. J Pathol 214: 149–160. 18161752 10.1002/path.2287

[2] Hehlgans T, Pfeffer K (2005) The intriguing biology of the tumour necrosis factor/tumour necrosis factor receptor superfamily: players, rules and the games. Immunology 115: 1–20. 15819693 10.1111/j.1365-2567.2005.02143.x PMC1782125

[3] Karin M, Gallagher E (2009) TNFR signaling: ubiquitin-conjugated TRAFfic signals control stop-and-go for MAPK signaling complexes. Immunol Rev 228: 225–240. 19290931 10.1111/j.1600-065X.2008.00755.x

[4] Schneider-Brachert W, Tchikov V, Neumeyer J, Jakob M, Winoto-Morbach S, et al (2004) Compartmentalization of TNF receptor 1 signaling: internalized TNF receptosomes as death signaling vesicles. Immunity 21: 415–428. 15357952 10.1016/j.immuni.2004.08.017

[5] Tsujimoto M, Yip YK, Vilcek J (1985) Tumor necrosis factor: specific binding and internalization in sensitive and resistant cells. Proc Natl Acad Sci U S A 82: 7626–7630. 2999773 10.1073/pnas.82.22.7626 PMC391386

[6] Schutze S, Tchikov V, Schneider-Brachert W (2008) Regulation of TNFR1 and CD95 signalling by receptor compartmentalization. Nat Rev Mol Cell Biol 9: 655–662. 18545270 10.1038/nrm2430

[7] Mahul-Mellier AL, Strappazzon F, Petiot A, Chatellard-Causse C, Torch S, et al (2008) Alix and ALG-2 are involved in tumor necrosis factor receptor 1-induced cell death. J Biol Chem 283: 34954–34965. 18936101 10.1074/jbc.M803140200 PMC3259881

[8] Dodeller F, Gottar M, Huesken D, Iourgenko V, Cenni B (2008) The lysosomal transmembrane protein 9B regulates the activity of inflammatory signaling pathways. J Biol Chem 283: 21487–21494. 18541524 10.1074/jbc.M801908200

[9] Liao W, Xiao Q, Tchikov V, Fujita K, Yang W, et al (2008) CARP-2 is an endosome-associated ubiquitin ligase for RIP and regulates TNF-induced NF-kappaB activation. Curr Biol 18: 641–649. 18450452 10.1016/j.cub.2008.04.017 PMC2587165

[10] Woo CH, Kim TH, Choi JA, Ryu HC, Lee JE, et al (2006) Inhibition of receptor internalization attenuates the TNFalpha-induced ROS generation in non-phagocytic cells. Biochem Biophys Res Commun 351: 972–978. 17097052 10.1016/j.bbrc.2006.10.154

[11] Jahner D, Hunter T (1991) The ras-related gene rhoB is an immediate-early gene inducible by v-Fps, epidermal growth factor, and platelet-derived growth factor in rat fibroblasts. Mol Cell Biol 11: 3682–3690. 1710770 10.1128/mcb.11.7.3682-3690.1991 PMC361128

[12] Fritz G, Kaina B, Aktories K (1995) The ras-related small GTP-binding protein RhoB is immediate-early inducible by DNA damaging treatments. J Biol Chem 270: 25172–25177. 7559652 10.1074/jbc.270.42.25172

[13] Kajimoto H, Hashimoto K, Bonnet SN, Haromy A, Harry G, et al (2007) Oxygen activates the Rho/Rho-kinase pathway and induces RhoB and ROCK-1 expression in human and rabbit ductus arteriosus by increasing mitochondria-derived reactive oxygen species: a newly recognized mechanism for sustaining ductal constriction. Circulation 115: 1777–1788. 17353442 10.1161/CIRCULATIONAHA.106.649566

[14] Wheeler AP, Ridley AJ (2004) Why three Rho proteins? RhoA, RhoB, RhoC, and cell motility. Exp Cell Res 301: 43–49. 15501444 10.1016/j.yexcr.2004.08.012

[15] Adamson P, Paterson HF, Hall A (1992) Intracellular localization of the P21rho proteins. J Cell Biol 119: 617–627. 1383236 10.1083/jcb.119.3.617 PMC2289677

[16] Gampel A, Parker PJ, Mellor H (1999) Regulation of epidermal growth factor receptor traffic by the small GTPase rhoB. Curr Biol 9: 955–958. 10508588 10.1016/s0960-9822(99)80422-9

[17] Huang M, Duhadaway JB, Prendergast GC, Laury-Kleintop LD (2007) RhoB regulates PDGFR-beta trafficking and signaling in vascular smooth muscle cells. Arterioscler Thromb Vasc Biol 27: 2597–2605. 17951322 10.1161/ATVBAHA.107.154211 PMC4384698

[18] Lajoie-Mazenc I, Tovar D, Penary M, Lortal B, Allart S, et al (2008) MAP1A light chain-2 interacts with GTP-RhoB to control epidermal growth factor (EGF)-dependent EGF receptor signaling. J Biol Chem 283: 4155–4164. 18056259 10.1074/jbc.M709639200

[19] Neel NF, Lapierre LA, Goldenring JR, Richmond A (2007) RhoB plays an essential role in CXCR2 sorting decisions. J Cell Sci 120: 1559–1571. 17405813 10.1242/jcs.03437 PMC2766565

[20] Rondanino C, Rojas R, Ruiz WG, Wang E, Hughey RP, et al (2007) RhoB-dependent modulation of postendocytic traffic in polarized Madin-Darby canine kidney cells. Traffic 8: 932–949. 17547697 10.1111/j.1600-0854.2007.00575.x

[21] Sandilands E, Akbarzadeh S, Vecchione A, McEwan DG, Frame MC, et al (2007) Src kinase modulates the activation, transport and signalling dynamics of fibroblast growth factor receptors. EMBO Rep 8: 1162–1169. 17975556 10.1038/sj.embor.7401097 PMC2267225

[22] Sandilands E, Cans C, Fincham VJ, Brunton VG, Mellor H, et al (2004) RhoB and actin polymerization coordinate Src activation with endosome-mediated delivery to the membrane. Dev Cell 7: 855–869. 15572128 10.1016/j.devcel.2004.09.019

[23] Fernandez-Borja M, Janssen L, Verwoerd D, Hordijk P, Neefjes J (2005) RhoB regulates endosome transport by promoting actin assembly on endosomal membranes through Dia1. J Cell Sci 118: 2661–2670. 15944396 10.1242/jcs.02384

[24] Wallar BJ, Deward AD, Resau JH, Alberts AS (2007) RhoB and the mammalian Diaphanous-related formin mDia2 in endosome trafficking. Exp Cell Res 313: 560–571. 17198702 10.1016/j.yexcr.2006.10.033

[25] Garcia-Mata R, Wennerberg K, Arthur WT, Noren NK, Ellerbroek SM, et al (2006) Analysis of activated GAPs and GEFs in cell lysates. Methods Enzymol 406: 425–437. 16472675 10.1016/S0076-6879(06)06031-9

[26] van der Pouw Kraan TC, Boeije LC, de Groot ER, Stapel SO, Snijders A, et al (1997) Reduced production of IL-12 and IL-12-dependent IFN-gamma release in patients with allergic asthma. J Immunol 158: 5560–5565. 9164981

[27] Engel ME, Datta PK, Moses HL (1998) RhoB is stabilized by transforming growth factor beta and antagonizes transcriptional activation. J Biol Chem 273: 9921–9926. 9545335 10.1074/jbc.273.16.9921

[28] Fritz G, Kaina B (2001) Transcriptional activation of the small GTPase gene rhoB by genotoxic stress is regulated via a CCAAT element. Nucleic Acids Res 29: 792–798. 11160903 10.1093/nar/29.3.792 PMC30383

[29] Westmark CJ, Bartleson VB, Malter JS (2005) RhoB mRNA is stabilized by HuR after UV light. Oncogene 24: 502–511. 15543229 10.1038/sj.onc.1208224

[30] Kishore N, Sommers C, Mathialagan S, Guzova J, Yao M, et al (2003) A selective IKK-2 inhibitor blocks NF-kappa B-dependent gene expression in interleukin-1 beta-stimulated synovial fibroblasts. J Biol Chem 278: 32861–32871. 12813046 10.1074/jbc.M211439200

[31] Adini I, Rabinovitz I, Sun JF, Prendergast GC, Benjamin LE (2003) RhoB controls Akt trafficking and stage-specific survival of endothelial cells during vascular development. Genes Dev 17: 2721–2732. 14597666 10.1101/gad.1134603 PMC280621

[32] Canguilhem B, Pradines A, Baudouin C, Boby C, Lajoie-Mazenc I, et al (2005) RhoB protects human keratinocytes from UVB-induced apoptosis through epidermal growth factor receptor signaling. J Biol Chem 280: 43257–43263. 16278215 10.1074/jbc.M508650200

[33] Mannam P, Zhang X, Shan P, Zhang Y, Shinn AS, et al (2013) Endothelial MKK3 is a critical mediator of lethal murine endotoxemia and acute lung injury. J Immunol 190: 1264–1275. 23275604 10.4049/jimmunol.1202012 PMC3552142

[34] Su X, Ao L, Zou N, Song Y, Yang X, et al (2008) Post-transcriptional regulation of TNF-induced expression of ICAM-1 and IL-8 in human lung microvascular endothelial cells: an obligatory role for the p38 MAPK-MK2 pathway dissociated with HSP27. Biochim Biophys Acta 1783: 1623–1631. 18486623 10.1016/j.bbamcr.2008.04.009 PMC2559815

[35] Llado A, Timpson P, Vila de MS, Moreto J, Pol A, et al (2008) Protein kinase Cdelta and calmodulin regulate epidermal growth factor receptor recycling from early endosomes through Arp2/3 complex and cortactin. Mol Biol Cell 19: 17–29. 17959830 10.1091/mbc.E07-05-0411 PMC2174165

[36] Rolfe BE, Worth NF, World CJ, Campbell JH, Campbell GR (2005) Rho and vascular disease. Atherosclerosis 183: 1–16. 15982657 10.1016/j.atherosclerosis.2005.04.023

[37] Kim BK, Kim HM, Chung KS, Kim DM, Park SK, et al (2011) Upregulation of RhoB via c-Jun N-terminal kinase signaling induces apoptosis of the human gastric carcinoma NUGC-3 cells treated with NSC12618. Carcinogenesis 32: 254–261. 21084431 10.1093/carcin/bgq244

[38] Kim DM, Won M, Chung CS, Kim S, Yim HJ, et al (2010) JNK-mediated transcriptional upregulation of RhoB is critical for apoptosis of HCT-116 colon cancer cells by a novel diarylsulfonylurea derivative. Apoptosis 15: 1540–1548. 20683666 10.1007/s10495-010-0531-7

[39] Kamon H, Kawabe T, Kitamura H, Lee J, Kamimura D, et al (2006) TRIF-GEFH1-RhoB pathway is involved in MHCII expression on dendritic cells that is critical for CD4 T-cell activation. EMBO J 25: 4108–4119. 16917499 10.1038/sj.emboj.7601286 PMC1560350

[40] Nwariaku FE, Rothenbach P, Liu Z, Zhu X, Turnage RH, et al (2003) Rho inhibition decreases TNF-induced endothelial MAPK activation and monolayer permeability. J Appl Physiol 95: 1889–1895. 12844496 10.1152/japplphysiol.00225.2003

[41] Teramoto H, Crespo P, Coso OA, Igishi T, Xu N, et al (1996) The small GTP-binding protein rho activates c-Jun N-terminal kinases/stress-activated protein kinases in human kidney 293T cells. Evidence for a Pak-independent signaling pathway. J Biol Chem 271: 25731–25734. 8824197 10.1074/jbc.271.42.25731

[42] Kotlyarov A, Neininger A, Schubert C, Eckert R, Birchmeier C, et al (1999) MAPKAP kinase 2 is essential for LPS-induced TNF-alpha biosynthesis. Nat Cell Biol 1: 94–97. 10559880 10.1038/10061

[43] Kyriakis JM, Avruch J (2001) Mammalian mitogen-activated protein kinase signal transduction pathways activated by stress and inflammation. Physiol Rev 81: 807–869. 11274345 10.1152/physrev.2001.81.2.807

[44] Lasa M, Mahtani KR, Finch A, Brewer G, Saklatvala J, et al (2000) Regulation of cyclooxygenase 2 mRNA stability by the mitogen-activated protein kinase p38 signaling cascade. Mol Cell Biol 20: 4265–4274. 10825190 10.1128/mcb.20.12.4265-4274.2000 PMC85794

[45] Neininger A, Kontoyiannis D, Kotlyarov A, Winzen R, Eckert R, et al (2002) MK2 targets AU-rich elements and regulates biosynthesis of tumor necrosis factor and interleukin-6 independently at different post-transcriptional levels. J Biol Chem 277: 3065–3068. 11741878 10.1074/jbc.C100685200

[46] Saklatvala J, Dean J, Clark A (2003) Control of the expression of inflammatory response genes. Biochem Soc Symp 95–106. 10.1042/bss0700095 14587285

[47] Winzen R, Kracht M, Ritter B, Wilhelm A, Chen CY, et al (1999) The p38 MAP kinase pathway signals for cytokine-induced mRNA stabilization via MAP kinase-activated protein kinase 2 and an AU-rich region-targeted mechanism. EMBO J 18: 4969–4980. 10487749 10.1093/emboj/18.18.4969 PMC1171568

[48] Wysk M, Yang DD, Lu HT, Flavell RA, Davis RJ (1999) Requirement of mitogen-activated protein kinase kinase 3 (MKK3) for tumor necrosis factor-induced cytokine expression. Proc Natl Acad Sci U S A 96: 3763–3768. 10097111 10.1073/pnas.96.7.3763 PMC22368

[49] Delcroix JD, Valletta JS, Wu C, Hunt SJ, Kowal AS, et al (2003) NGF signaling in sensory neurons: evidence that early endosomes carry NGF retrograde signals. Neuron 39: 69–84. 12848933 10.1016/s0896-6273(03)00397-0

[50] Pelkmans L, Zerial M (2005) Kinase-regulated quantal assemblies and kiss-and-run recycling of caveolae. Nature 436: 128–133. 16001074 10.1038/nature03866

[51] Miaczynska M, Pelkmans L, Zerial M (2004) Not just a sink: endosomes in control of signal transduction. Curr Opin Cell Biol 16: 400–406. 15261672 10.1016/j.ceb.2004.06.005

[52] Bradley JR, Johnson DR, Pober JS (1993) Four different classes of inhibitors of receptor-mediated endocytosis decrease tumor necrosis factor-induced gene expression in human endothelial cells. J Immunol 150: 5544–5555. 8390537

