# Supplementary figures and images for: The Small GTPase RhoB Regulates TNFα Signaling in Endothelial Cells
Source: PLoS One. 2013 Sep 26;8(9):e75031. doi: 10.1371/journal.pone.0075031 (PMC3784429; doi:10.1371/journal.pone.0075031)

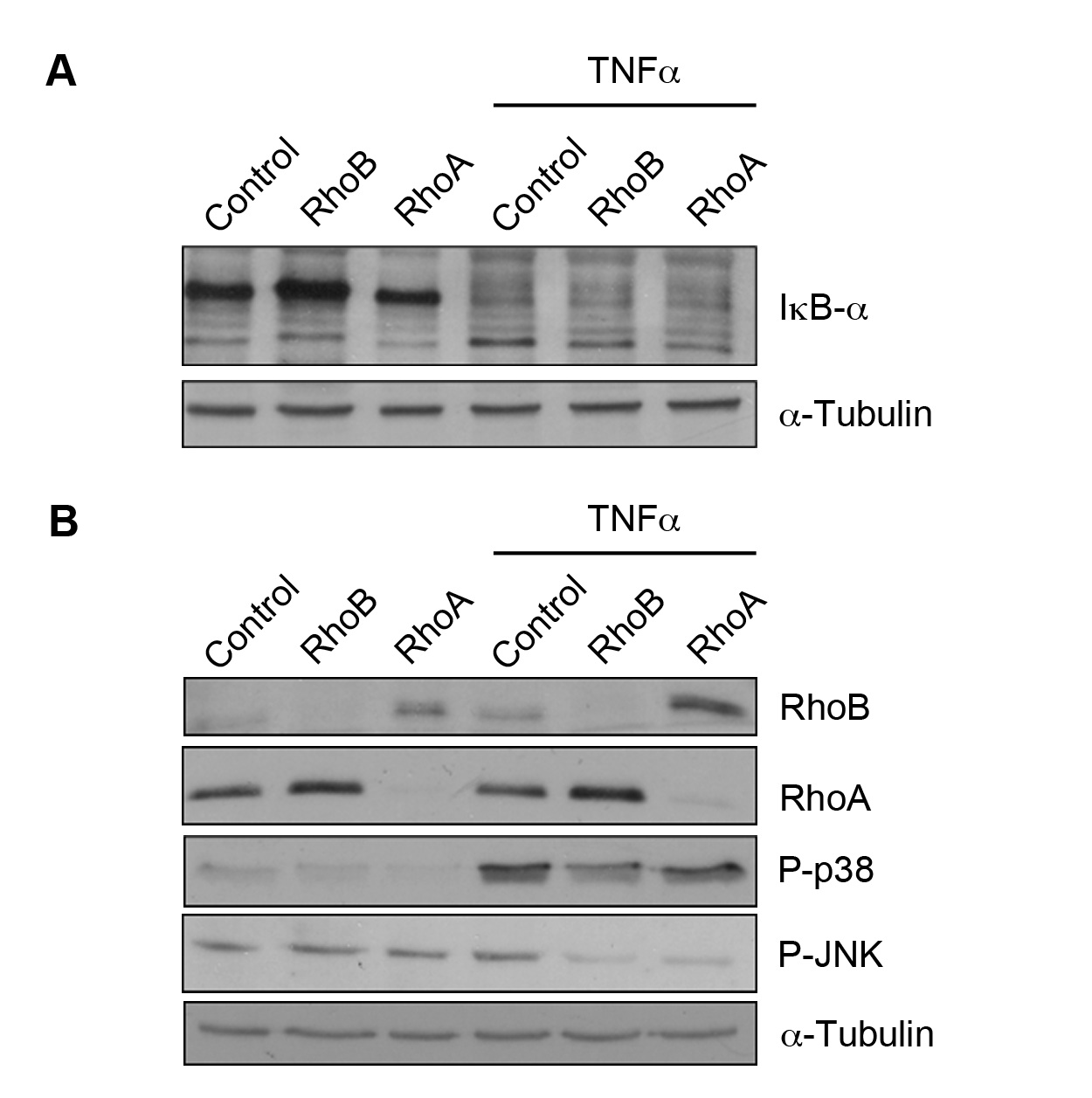

Supplement: Figure S1 — (A) Cells transfected with a pool of 3 RhoB siRNAs, with a RhoA siRNA or with siRNA control were stimulated or not with TNFα for 30 minutes and IκBα was detected by western blotting of cell lysates. α-Tubulin was detected as control for equal protein loading; (B) Cells transfected with siRNAs mentioned above were stimulated with TNFα for 30 minutes. Subsequently, phospho-p38 and phospho-JNK were detected by western blotting of cell lysates. α-Tubulin was detected as control for equal protein loading. (TIF) [file pone.0075031.s001.tif]
